# Applying an Open-Source Segmentation Algorithm to Different OCT Devices in Multiple Sclerosis Patients and Healthy Controls: Implications for Clinical Trials

**DOI:** 10.1155/2015/136295

**Published:** 2015-05-18

**Authors:** Pavan Bhargava, Andrew Lang, Omar Al-Louzi, Aaron Carass, Jerry Prince, Peter A. Calabresi, Shiv Saidha

**Affiliations:** ^1^Department of Neurology, Johns Hopkins University School of Medicine, Baltimore, MD 21287, USA; ^2^Department of Electrical and Computer Engineering, Johns Hopkins University, Baltimore, MD 21287, USA

## Abstract

*Background*. The lack of segmentation algorithms operative across optical coherence tomography (OCT) platforms hinders utility of retinal layer measures in MS trials. *Objective*. To determine cross-sectional and longitudinal agreement of retinal layer thicknesses derived from an open-source, fully-automated, segmentation algorithm, applied to two spectral-domain OCT devices. *Methods*. Cirrus HD-OCT and Spectralis OCT macular scans from 68 MS patients and 22 healthy controls were segmented. A longitudinal cohort comprising 51 subjects (mean follow-up: 1.4 ± 0.9 years) was also examined. Bland-Altman analyses and interscanner agreement indices were utilized to assess agreement between scanners. *Results*. Low mean differences (−2.16 to 0.26 *μ*m) and narrow limits of agreement (LOA) were noted for ganglion cell and inner and outer nuclear layer thicknesses cross-sectionally. Longitudinally we found low mean differences (−0.195 to 0.21 *μ*m) for changes in all layers, with wider LOA. Comparisons of rate of change in layer thicknesses over time revealed consistent results between the platforms. *Conclusions*. Retinal thickness measures for the majority of the retinal layers agree well cross-sectionally and longitudinally between the two scanners at the cohort level, with greater variability at the individual level. This open-source segmentation algorithm enables combining data from different OCT platforms, broadening utilization of OCT as an outcome measure in MS trials.

## 1. Introduction

Multiple sclerosis (MS) is a chronic demyelinating disorder of the central nervous system, with both inflammatory and neurodegenerative components [1]. MS has a predilection to affect the optic nerves with autopsy studies revealing that up to 99% of MS patients have involvement of the optic nerves, regardless of optic neuritis history [2–4]. While acute demyelination and inflammatory axonal transection may be responsible for the symptoms observed during an acute relapse, neuroaxonal degeneration appears to be the principal pathological substrate underlying accumulation of disability and progression in MS [5–7]. Several putative therapeutic strategies for remyelination and neuroprotection are now transitioning from the laboratory to early phase clinical trials [8–10]. The anterior visual pathway has been proposed as an ideal model within which to study the effect of such therapies, due to its excellent structure-function correlations [11, 12].

Optical coherence tomography (OCT) is a rapid, noninvasive, well tolerated, and reproducible method utilizing low-coherence, near-infrared light to generate high-resolution, cross-sectional images of the retina [13]. Advances in OCT technology have led to shorter scan times, improved resolution, and better reproducibility [14, 15]. Current generation spectral-domain (SD) OCT devices have a resolution of approximately 4-5 *μ*m. Initial studies of OCT in MS primarily focused on peripapillary retinal nerve fiber layer (p-RNFL) and total macular volume (TMV) measurements [16, 17]. Recently developed automated retinal layer segmentation algorithms have enabled examination of alterations within discrete retinal layers in MS [18–20]. Optic nerve pathology results in degeneration of its constituent axons, the retinal nerve fiber layer, and ganglion cell neurons, from which optic nerve axons derive. Moreover, studies suggest that primary retinal pathology may also be operative in MS, though this has been challenged by other studies [20, 21]. Previous studies found that increased inner nuclear layer (INL) thickness may be associated with the development of new T2 lesions, contrast enhancing lesions, and EDSS progression, while p-RNFL and ganglion cell layer (GCL) thicknesses may correlate with grey matter volume [22, 23]. Despite these findings and the relative inexpensiveness of OCT, OCT derived measures have not been widely employed as outcome measures in clinical trials. This likely relates to the utilization of different OCT platforms across varying clinical sites and the fact that currently employed segmentation algorithms are mostly platform specific. This is a barrier for not only MS research and trials, but also virtually all disciplines in which OCT is of interest. The comparison of quantitative results across OCT platforms has been a challenge, since manufacturer segmentation algorithms utilize different anatomical landmarks from which retinal measures are calculated [24, 25]. An open-source segmentation algorithm that could be used to segment OCT scans from different OCT platforms in a consistent fashion could allow more widespread use of OCT in clinical trials, provided the agreement between acquired measures across the platforms was good. In this study comprising cross-sectional and longitudinal cohorts, we performed a cross-platform comparison of retinal layer OCT segmentation utilizing a new open-source segmentation algorithm and also compared derived measures between MS patients and healthy controls.

## 2. Methods

### 2.1. Study Population

Patients for this study were recruited from the Johns Hopkins Multiple Sclerosis Center by convenience sampling. Written informed consent was obtained from study participants. The study was approved by the Institutional Review Board of Johns Hopkins University, was HIPAA compliant, and adhered to the tenets laid down in the Declaration of Helsinki. MS diagnosis and subtype classification into relapsing-remitting (RRMS), secondary progressive (SPMS), or primary progressive (PPMS) were confirmed by the treating neurologist, based on the revised McDonald criteria [26]. Healthy controls (HCs) were recruited from volunteers amongst Johns Hopkins staff. Individuals with refractive errors of > ±6.0 diopters, history of ocular surgery, glaucoma, hypertension, diabetes, or any other apparent ocular pathology were excluded.

### 2.2. OCT Scanning

Retinal imaging was performed by experienced technicians on Cirrus HD-OCT model 4000, software version 5.0 (Carl Zeiss Meditec, Dublin, CA, USA) and Spectralis OCT, software version 5.2.4 (Heidelberg Engineering, Heidelberg, Germany), as described in detail elsewhere [15, 25]. Briefly, Cirrus macular data was obtained using the macular cube 512 × 128 protocol. OSCAR-1B quality control criteria were applied to OCT scans. Only scans with signal strengths ≥ 7 and without artifact were included in the study. Spectralis macular scans were obtained using the fast macular protocol. Spectralis macular scans included in this study had an automatic real time (ART) of 16 and signal strength ≥ 20 dB and were devoid of artifact. We removed 8 scans that did not fulfill these quality control criteria. Cirrus and Spectralis scans were obtained in a random order on the same day.

### 2.3. OCT Segmentation

**Figure 1:**
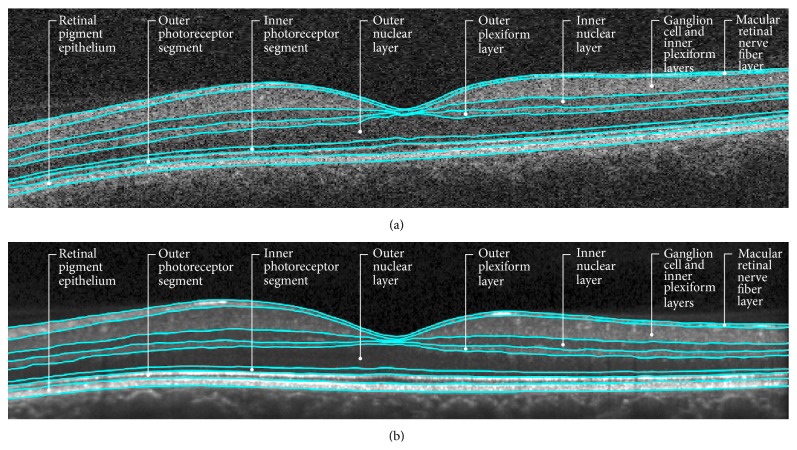
Depiction of retinal segmentation of concomitant Cirrus HD-OCT and Spectralis scans. This figure demonstrates the retinal layer segmentation in corresponding scans from a patient with MS, acquired on Cirrus HD-OCT (a) and Spectralis (b) scanners. The different retinal layers segmented by the automated segmentation algorithm have been labeled.

Layer segmentation of the OCT data was performed using a previously developed and validated algorithm for detecting 8 layers within the macula as depicted in Figure 1 [27]. The algorithm works in three stages: preprocessing, pixel classification, and graph-based multilayer segmentation. In the preprocessing stage, the intensities of each B-scan image are normalized to add consistency between scans. Additionally, estimates of the inner and outer retinal boundaries (inner limiting membrane (ILM) and Bruch's membrane (BM)) are used to restrict the region of interest for the algorithm, as well as to flatten the data to the BM boundary. In the second stage, we use a random forest classifier to determine the probability that each pixel belongs to one of the 9 layer boundaries [28]. The classifier was trained using manual segmentations from 7 randomly chosen subjects (including both MS and control data). In the final stage, a graph-based segmentation algorithm is used to find the 9 surfaces (corresponding to the boundaries between each of the 8 layers) by maximizing the boundary-specific probabilities on those surfaces [29]. Constraints are used to limit the minimum and maximum distance between each boundary and to limit the smoothness of the final segmentation. Thickness measurements are computed by averaging the thickness values within a square 5 × 5 mm region centered at the fovea. The center of the fovea was estimated as the location of the A-scan having the smallest total macular thickness within the central 2 × 2 mm area of the data. Note that the thickness values were not averaged over the entire 6 × 6 mm imaged area since we allow the position of the fovea to vary by ±1 mm. This algorithm is available for download at http://www.nitrc.org/projects/aura_tools/. The run time for the algorithm is between 3 and 4 minutes per scan. Cirrus scans were exported in  .img format prior to segmentation with the algorithm, while Spectralis scans were exported in  .vol format. Following segmentation, scans were inspected for segmentation errors. The segmentation software allows for scans that have segmentation errors identified on visual inspection to have segmentation lines corrected manually.

The layers produced by manufacturer segmentation algorithms include mRNFL, ganglion cell + inner plexiform layer (GCIP), inner nuclear layer + outer plexiform layer (INL + OPL), and outer nuclear layer + photoreceptor (ONL + PR) for the Cirrus HD-OCT and mRNFL, GCL, IPL, INL, OPL, ONL, photoreceptor-inner segment (PR-IS), photoreceptor-outer segment (PR-OS), and retinal pigment epithelium (RPE) for Spectralis.

### 2.4. Statistical Analysis

Statistical analyses were performed using Stata 13 (Stata-Corp, College Station, TX). We utilized Bland-Altman analyses and interscanner agreement indices to compare the retinal layer thickness measures derived from the two OCT platforms [30, 31]. This included calculating mean differences with 95% confidence intervals (CI), limits of agreement (LOA) with 95% CI, and Bland-Altman plots of differences against average measurements. These were calculated for all retinal layers. The interscanner agreement index was calculated for each retinal layer for each subject. This index has previously been used to compare interscanner variation between MRI platforms [31], as well as OCT measures derived from different scanners [18]. If *X*_*a*_ is the measurement on machine *a*, and *X*_*b*_ is the measurement on machine *b*, then the interscanner agreement is defined as follows:

(1) $$\begin{array}{c} \text{Interscanner agreement index}=1-\frac{\left|X_{a}-X_{b}\right|}{\left(X_{a}+X_{b}\right)/2}. \end{array}$$

For the longitudinal cohort we performed modified Bland-Altman analysis to adjust for repeated measures, utilizing the change in various retinal layers between serial scans [32]. Similar to the cross-sectional cohort, we calculated mean differences and LOA with 95% CI and Bland-Altman plots. We utilized mixed effects linear models to calculate the rate of change of layer thickness over time, adjusting for age and sex and accounting for within-subject intereye correlations.

We performed an exploratory comparison of thicknesses of various retinal layers (derived from each platform) between MS subjects and healthy controls utilizing a mixed effects linear regression model, adjusting for age and sex, accounting for within-subject intereye correlations. *p* values < 0.05 were defined as statistically significant.

## 3. Results

### 3.1. Study Population

The cross-sectional cohort consisted of 90 subjects, 68 MS patients and 22 HCs. The longitudinal cohort was a subgroup of the cross-sectional cohort, consisting of 51 subjects. The demographic characteristics of the study participants are illustrated in Table 1. MS patients were significantly older than HCs (*p* < 0.001), with an insignificantly greater proportion of MS patients being female (*p* = 0.25). The mean follow-up duration of the longitudinal cohort was 1.4 ± 0.9 years.

### 3.2. Cross-Sectional Comparison of Segmentation across OCT Platforms

**Figure 2:**
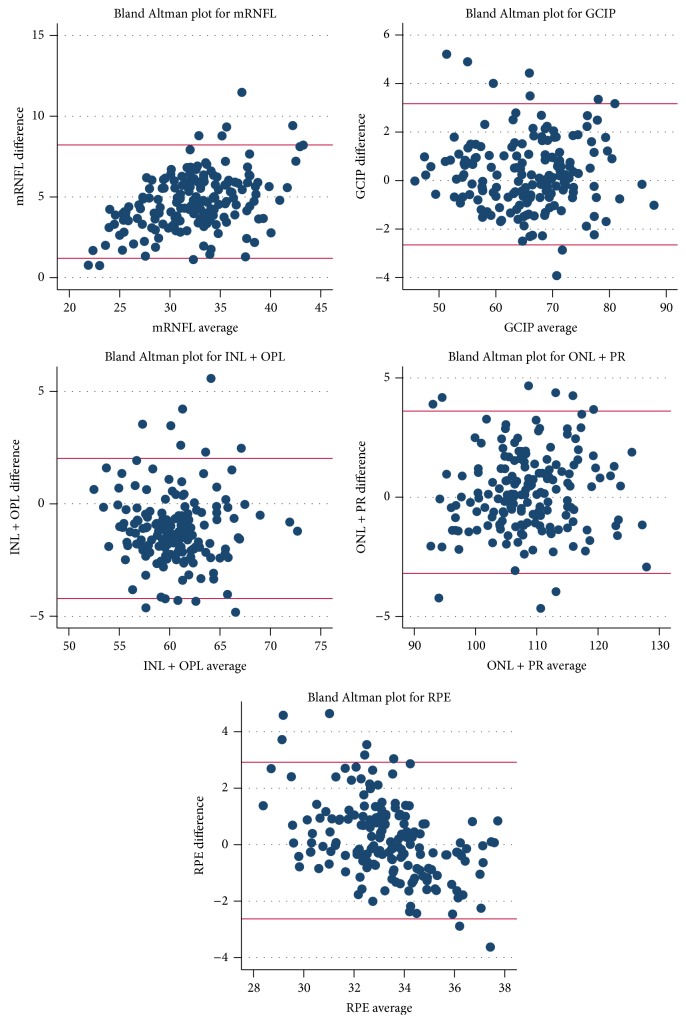
Bland-Altman plots of cross-sectional comparison of retinal layer segmentation. Scatter plots of the difference in measurement between the two scanners versus average of measurement from two scanners. The horizontal lines represent the upper and lower limits of agreement (LOA). The majority of plotted points fall within the LOA as expected in all plots.

Comparing Spectralis to Cirrus, Bland-Altman analyses revealed low mean differences for ganglion cell layer + inner plexiform layer (GCIP): 0.26 *μ*m, inner nuclear layer (INL): −1.31 *μ*m, inner nuclear layer + outer plexiform layer (INL + OPL): −1.09 *μ*m, outer nuclear layer + photoreceptor segments (ONL + PR): 0.20 *μ*m, and retinal pigment epithelium (RPE): 0.14 *μ*m thicknesses. A slightly larger mean difference was noted for the outer nuclear layer (ONL) thickness by itself: −2.16 *μ*m, with the largest mean difference seen in the macular-retinal nerve fiber layer (mRNFL) thickness: 5.11 *μ*m. This suggests excellent agreement at the cohort level for all retinal layer measures except for the ONL and mRNFL. The limits of agreement (LOA) were relatively narrow for all retinal layers considering the thicknesses of the individual layers (measurements in *μ*m, mRNFL LOA: 1.86, 8.34, GCIP LOA: −2.65, 3.17, INL + OPL LOA: −4.21, 2.02, and ONL + PR LOA: −3.20, 3.61). The mean differences, 95% CI for mean differences, and LOA for all layers are listed in Table 2. The mean differences and LOA for all retinal layers were similar between MS patients and healthy controls (data not shown). We also constructed Bland-Altman plots for these comparisons for each layer (Figure 2). These showed no evidence of a systematic relationship between differences and average thickness values.

**Figure 3:**
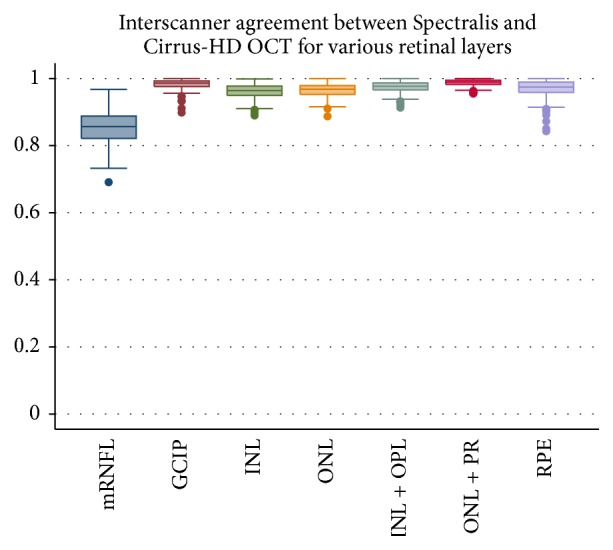
Interscanner agreement indices comparing Spectralis and Cirrus HD-OCT. The graph shows box plots of interscanner agreement indices for individual retinal layers. Except for the macular retinal nerve fiber layer (mRNFL), all other layers show excellent agreement evidenced by values close to 1.0.

Interscanner agreement indices were extremely high for all layers except for the mRNFL (mRNFL: 85.5 ± 4.9%, GCIP: 98.3 ± 1.5%, INL: 96.2 ± 2.0%, INL + OPL: 97.4 ± 1.7%, ONL: 96.4 ± 2.1%, ONL + PR: 98.7 ± 1.0%, and RPE: 96.8 ± 2.8%). The box plots of the interscanner agreement indices for various layers are depicted in Figure 3. These results further support excellent agreement between OCT segmentation measures across the cohort between the two scanners.

### 3.3. Longitudinal Comparison of Segmentation across Platforms

**Figure 4:**
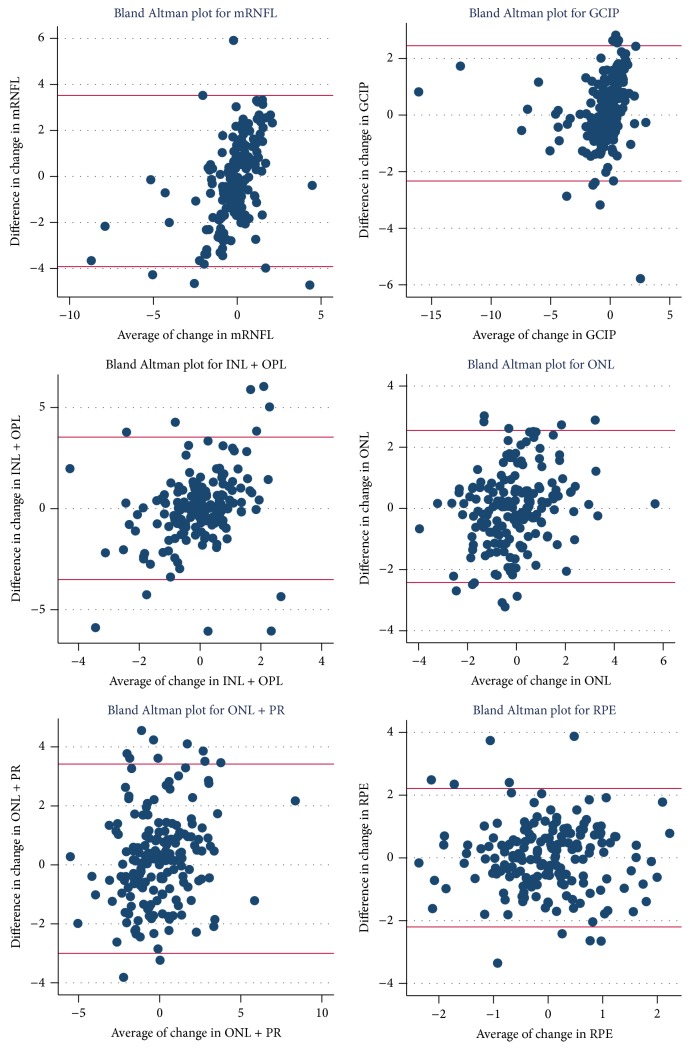
Bland-Altman plots of longitudinal comparison of change in retinal layer thicknesses. Scatter plots of the difference versus the average of change in the retinal layer measurements from two scanners. The horizontal lines represent the upper and lower limits of agreement (LOA). The majority of plotted points fall within the LOA as expected in all plots.

Using a modified Bland-Altman analysis we compared the changes in layer thicknesses over time, between scanners. We found small mean differences for changes in all layers, mRNFL: −0.19 *μ*m, GCIP: 0.06 *μ*m, INL + OPL: 0.015 *μ*m, ONL: 0.016 *μ*m, ONL + PR: 0.21 *μ*m, and RPE: 0.009 *μ*m. The LOA for the change in various retinal layers in the longitudinal cohort were numerically similar to those from the cross-sectional cohort; however, compared to the absolute values of change in these layers derived from the two scanners, these LOA appeared larger, suggesting poor agreement at the individual level across the scanners for change in layer thickness over time (mRNFL LOA: −3.92, 3.52, GCIP LOA: −2.33, 2.45, INL + OPL LOA: −3.51, 3.54, ONL LOA: −2.52, 2.55, ONL + PR LOA: −3.0, 3.42, and RPE LOA: −2.2, 2.21). The mean differences, 95% CI for mean differences, and LOA for all layers are listed in Table 3. Bland-Altman plots for the change in retinal layers across the two devices are shown in Figure 4.

We also utilized mixed effects models to ascertain the rate of change of different retinal layers for the entire cohort using segmentation values derived from the two platforms. These analyses are summarized in Table 4. We found that, except for the mRNFL, the remaining layers showed consistency in the significance of rate of change in the layer thicknesses between the two platforms.

### 3.4. Comparison of Retinal Layers between MS and Healthy Controls

In the cross-sectional cohort, relative to controls, MS patients had reduced mRNFL (*p* = 0.001) and GCIP (*p* < 0.001) thicknesses across both platforms, adjusting for age and gender.  Table 5 lists difference in the mean thickness values of individual layers between HCs and MS subjects, with separate comparisons for each OCT platform.

## 4. Discussion

The results of this study reveal excellent agreement of retinal layer measures acquired from two different OCT scanners, both cross-sectionally and longitudinally across MS patients and healthy controls, helping to validate a new automated retinal segmentation algorithm operative across platforms. Utilizing this segmentation technique could help overcome current limitations in comparing retinal segmentation data across different OCT scanners, enabling wider adoption of OCT measures as outcomes in clinical trials.

The results of the cross-sectional comparison suggest excellent cross-platform agreement at the cohort level for the GCIP, INL, INL + OPL, and ONL + PR, as evidenced by the small mean differences for these measures between the two OCT devices studied. The mean difference for the mRNFL was larger suggesting poorer agreement between scanners. The small mean differences suggest that at the cohort level retinal layer measures are comparable across platforms. This is an important finding since it suggests data acquired using different scanners could be pooled, utilizing this segmentation algorithm. In multicenter studies, where different sites may have different scanners, this could allow an increase in sample size, power, and ultimately the ability to detect meaningful relationships between OCT and other clinical and imaging measures of MS disease activity. Further support for agreement is derived from our analyses comparing retinal layer thickness measures between MS patients and healthy controls acquired from the two different OCT scanners. These results were consistent in terms of magnitude of difference, as well as significance, across both platforms for all retinal neuronal layers, underpinning the potential utility of employing a consistent segmentation algorithm for the examination of cross-sectional data acquired on different OCT scanners. Similar results were obtained in the longitudinal cohort suggesting that utilizing retinal layer thicknesses from two different OCT platforms may not change the interpretation of the rates of layer change across the entire cohort.

The LOA represent the agreement of measures at an individual level. Although we found the LOA to be narrower than those reported in previous studies, they may still be unacceptably wide to support the use of different platforms interchangeably at the individual patient level. In routine clinical practice, therefore, patients should continue to be scanned on the same OCT device, as has been suggested in prior studies [18, 25].

Results from the longitudinal cohort revealed small mean differences for all retinal layers. Comparing these mean differences to the absolute values for change in the layers, the GCIP, INL + OPL, ONL, and RPE mean differences suggested good agreement at the cohort level. The mean differences for mRNFL, INL, and ONL + PR appeared large compared to the absolute values of the change in those layers. This suggests that the GCIP, INL + OPL, ONL, and RPE agreed well between the scanners at the cohort level over time, raising the possibility that the employed segmentation algorithm may have utility not only cross-sectionally, but longitudinally as well. Elaborating upon these findings, when assessing the rate of change of individual retinal layers over time (adjusting for age and gender), we found consistent results between the platforms in terms of the direction and significance of change in various retinal layer thicknesses with the exception of the mRNFL. This would suggest that, despite the mean differences being large compared to the absolute rate of change for some layers, combining data at the cohort level may be possible for all layers except for the mRNFL. Similar to the cross-sectional cohort, the LOA for the longitudinal cohort suggested poorer agreement at the individual level. Thus it would not be advisable to use different scanners while following up an individual patient over time.

TMV and pRNFL have been previously compared across scanners [24, 25, 33]. Some of these studies suggested poor agreement between different OCT platforms. The limitation with these studies is that manufacturers utilize different landmarks to calculate retinal thicknesses, thus making it difficult to compare across platforms. The use of a common algorithm to segment data from different platforms helps to circumvent this problem by utilizing consistent landmarks.

It has been suggested that there may be inherent limitations to comparing data from different scanners. A study comparing lateral and axial thickness measures derived from different SD-OCT scanners imaging a phantom eye showed significant differences between OCT platforms [34]. The authors suggested using a conversion factor when attempting to compare retinal measures across scanners. This study did not attempt to segment retinal layers and utilized manual rather than automated measurement methods. In contrast, another study utilizing manual delineation of retinal boundaries showed that it was possible to obtain almost identical retinal thickness values from different OCT scanners [35]. The use of an automated method may facilitate more consistent and accurate measurement of retinal layer thicknesses.

A limitation of this study is that the novel segmentation technique employed was only applied to two OCT platforms, and we therefore do not know whether its application to other OCT scanners may be as effective. Moreover, due to the novelty of this algorithm and the single center nature of this study, the findings of this study should be replicated in a larger sample size, over multiple centers, preferably incorporating more OCT platforms, and a wider host of neuroophthalmological/ophthalmological disorders to generate more generalizable and definitive results. Despite these limitations, this study represents a major advance in terms of demonstrating the utility and advantages of applying a consistent segmentation technique to scans acquired from different OCT scanners.

An important development that could help in the application of segmentation algorithms such as ours, as well as the incorporation of OCT images into patient electronic medical records, would be the development of DICOM standards that would be utilized by multiple OCT device manufacturers. This could help expand the application of novel segmentation algorithms to multiple OCT platforms and their use in OCT studies as well as clinical practice.

The availability of an open-source OCT segmentation algorithm would be of interest to those conducting observational studies utilizing OCT, allowing them to increase sample size and pool data across centers. Such a segmentation technique is even more critical for the incorporation of OCT as a routine outcome measure in trials. From an MS perspective this is important since a new wave of trials of remyelinating therapies is poised to begin. At present, we have limited techniques to measure the effects of such therapies in humans. OCT has been proposed as an important tool that could be specifically utilized for this purpose based on the supposition that rapid remyelination will protect retinal axons from degeneration. OCT could also be incorporated as an outcome measure in trials of putative neuroprotective agents. The ability to easily and consistently segment OCT scans, in order to derive reliable retinal segmentation data from different scanners at multiple centers, would be an essential prerequisite to increasing the utilization of OCT in not only these scenarios, but also a whole host of other research and trial settings. Therefore our validation of a novel retinal segmentation algorithm, which can be consistently applied across OCT platforms, could be a major step towards expanding OCT utilization in not only MS research, but also other neuroophthalmological disorders, ultimately facilitating therapeutic advances.

## Figures and Tables

**Table 1:** Demographic characteristics of subjects in the cross-sectional cohort.

Category	Number of subjects	Age (SD)	Sex ratio (female : male)	Disease duration (SD)
Healthy controls	22	33.5 (9.1)	13 : 9	N.A
RRMS	52	41.8 (11.6)	37 : 15	10.3 (7.3)
SPMS	9	59.5 (6.1)	8 : 1	27.6 (10.6)
PPMS	7	56.2 (6.4)	4 : 3	19.7 (12.9)

RRMS: relapsing-remitting multiple sclerosis; SPMS: secondary progressive multiple sclerosis; PPMS: primary progressive multiple sclerosis; SD: standard deviation.

**Table 2:** Cross-sectional comparison of retinal layer thickness between Spectralis and Cirrus HD-OCT.

Layer	Average thickness^∗^ (SD) (*μ*m)	Mean difference (95% CI) (*μ*m)	Upper LOA (95% CI) (*μ*m)	Lower LOA (95% CI) (*μ*m)
mRNFL	32.35 (4.38)	4.71 (4.45 to 4.97)	8.22 (7.77, 8.68)	1.20 (0.74, 1.65)
GCIP	65.59 (8.13)	0.26 (0.04 to 0.48)	3.17 (2.79, 3.55)	−2.65 (−3.03, −2.27)
INL	35.59 (1.81)	−1.31 (−1.43 to −1.12)	0.20 (0.01, 0.40)	−2.83 (−3.03, −2.63)
INL + OPL	60.59 (3.31)	−1.09 (−1.33 to −0.86)	2.02 (1.62, 2.43)	−4.21 (−4.62, −3.81)
ONL	63.10 (5.80)	−2.16 (−2.35, −1.97)	0.39 (0.05, 0.72)	−4.71 (−5.05, −4.38)
ONL + PR	108.56 (7.14)	0.20 (−0.05 to 0.46)	3.61 (3.17, 4.05)	−3.20 (−3.65, −2.76)
RPE	33.32 (1.92)	0.14 (−0.06 to 0.35)	2.92 (2.56, 3.28)	−2.63 (−2.99, −2.27)

^**∗**^Average of thickness values derived from both OCT platforms. mRNFL: macular retinal nerve fiber layer; GCIP: ganglion cell layer + inner plexiform layer; INL: inner nuclear layer; INL + OPL: inner nuclear layer + outer plexiform layer; ONL: outer nuclear layer; ONL + PR: outer nuclear layer + photoreceptor segments; RPE: retinal pigment epithelium.

**Table 3:** Longitudinal comparison of retinal layer thicknesses between Spectralis and Cirrus HD-OCT.

Layer	Mean change Spectralis (SD) (*μ*m)	Mean change Cirrus (SD) (*μ*m)	Mean difference (95% CI) (*μ*m)	Upper LOA (95% CI) (*μ*m)	Lower LOA (95% CI) (*μ*m)
mRNFL	−0.29 (2.11)	−0.09 (1.42)	−0.195 (−0.47 to 0.08)	3.52 (3.04, 4.00)	−3.92 (−4.40, −3.43)
GCIP	−0.54 (2.27)	−0.60 (2.16)	0.060 (−0.12 to 0.24)	2.45 (2.15, 2.76)	−2.33 (−2.64, −2.02)
INL	−0.046 (0.69)	−0.004 (0.71)	−0.042 (−0.16 to 0.07)	1.56 (1.36, 1.77)	−1.65 (−1.86, −1.44)
INL + OPL	−0.041 (1.59)	−0.056 (1.17)	0.015 (−0.25 to 0.28)	3.54 (3.09, 4.00)	−3.51 (−3.97, −3.06)
ONL	−0.073 (1.60)	−0.089 (1.33)	0.016 (−0.17 to 0.20)	2.55 (2.22, 2.87)	−2.52 (−2.85, −2.19)
ONL + PR	0.04 (2.20)	−0.17 (1.91)	0.213 (−0.03 to 0.45)	3.42 (3.01, 3.84)	−3.00 (−3.42, −2.59)
RPE	−0.023 (1.01)	−0.032 (1.05)	0.009 (−0.15 to 0.17)	2.21 (1.93, 2.50)	−2.20 (−2.49, −1.91)

mRNFL: macular retinal nerve fiber layer; GCIP: ganglion cell layer + inner plexiform layer; INL: inner nuclear layer; INL + OPL: inner nuclear layer + outer plexiform layer; ONL: outer nuclear layer; ONL + PR: outer nuclear layer + photoreceptor segments; RPE: retinal pigment epithelium.

**Table 4:** Comparison of the rates of change of retinal layer thickness between Spectralis and Cirrus HD-OCT.

Layer	Rate of change of layer thickness, Spectralis mean (95% CI) (*μ*m/year)	*p* value	Rate of change of layer thickness, Cirrus mean (95% CI) (*μ*m/year)	*p* value
mRNFL	−0.28 (−0.56, −0.001)	0.049	−0.09 (−0.28, 0.09)	0.326
GCIP	−0.59 (−0.89, −0.27)	<0.001	−0.66 (−0.96, −0.36)	<0.001
INL	−0.07 (−0.16, 0.01)	0.102	−0.06 (−0.16, 0.03)	0.198
INL + OPL	−0.08 (−0.27, 0.11)	0.419	−0.11 (−0.26, 0.04)	0.157
ONL	−0.06 (−0.25, 0.13)	0.526	−0.11 (−0.27, 0.05)	0.196
ONL + PR	0.05 (−0.21, 0.31)	0.689	−0.16 (−0.39, 0.07)	0.166
RPE	0.004 (−0.12, 0.12)	0.947	−0.002 (−0.13, 0.12)	0.971

mRNFL: macular retinal nerve fiber layer; GCIP: ganglion cell layer + inner plexiform layer; INL: inner nuclear layer; INL + OPL: inner nuclear layer + outer plexiform layer; ONL: outer nuclear layer; ONL + PR: outer nuclear layer + photoreceptor segments; RPE: retinal pigment epithelium.

**Table 5:** Cross-sectional comparison of retinal layer thickness between HCs and MS subjects.

Layer	Difference in mean thickness (Spectralis) (*μ*m)	*p* value^∗^	Difference in mean thickness (Cirrus) (*μ*m)	*p* value^∗^
mRNFL	−3.54	0.001	−3.62	<0.001
GCIP	−9.47	<0.001	−9.04	<0.001
INL	−0.57	0.241	−0.31	0.498
INL + OPL	−0.87	0.321	−0.66	0.459
ONL	−2.58	0.087	−2.02	0.154
ONL + PR	−2.71	0.14	−2.44	0.172
RPE	−1.39	0.001	−0.92	0.131

^∗^Values adjusted for age and gender. mRNFL: macular retinal nerve fiber layer; GCIP: ganglion cell layer + inner plexiform layer; INL: inner nuclear layer; INL + OPL: inner nuclear layer + outer plexiform layer; ONL: outer nuclear layer; ONL + PR: outer nuclear layer + photoreceptor segments; RPE: retinal pigment epithelium.
